# Efficacy and Safety of Paclitaxel-Coated Balloon Angioplasty in Patients With In-Stent Restenosis With vs Without Diabetes

**DOI:** 10.1016/j.jscai.2026.105320

**Published:** 2026-04-09

**Authors:** Prakriti Gaba, Brian K. Jefferson, Richard Shlofmitz, Jeffrey W. Moses, William Bachinsky, Suhail Dohad, Steven Rudick, Robert Stoler, William Nicholson, John Altman, Cinthia Bateman, Amar Krishnaswamy, Rafael Cavalcante, Robert W. Yeh, Ajay J. Kirtane

**Affiliations:** a Brigham and Women's Hospital, Boston, Massachusetts; b HCA Centennial Medical Center, Nashville, Tennessee; c St. Francis Hospital - The Heart Center, Roslyn, New York; d NewYork-Presbyterian Hospital/Columbia University Irving Medical Center, New York, New York; e Cardiovascular Research Foundation, New York, New York; f University of Pittsburgh Medical Center, Central PA, Harrisburg, Pennsylvania; g Cedars Sinai Medical Center, Los Angeles, California; h Lindner Center for Research and Education at The Christ Hospital, Cincinnati, Ohio; i Baylor Heart & Vascular Hospital, Dallas, Texas; j Emory University Hospital, Atlanta, Georgia; k St. Anthony Hospital, Denver, Colorado; l South Denver Cardiology, Littleton, Colorado; m Cleveland Clinic Foundation, Cleveland, Ohio; n Boston Scientific Corporation, Marlborough, Massachusetts; o Beth Israel Deaconess Medical Center, Boston, Massachusetts

**Keywords:** diabetes mellitus, in-stent restenosis, paclitaxel-coated balloon, target lesion failure, target lesion revascularization, uncoated balloon

## Abstract

**Background:**

The AGENT IDE trial demonstrated superiority of a paclitaxel-coated balloon over an uncoated balloon in treating patients with coronary in-stent restenosis (ISR). Whether the efficacy and safety of paclitaxel-coated vs uncoated balloon differ based on diabetes status remains unclear.

**Methods:**

A total of 600 patients with ISR were randomized (2:1) to paclitaxel-coated or uncoated balloon. The primary end point was target lesion failure (TLF), which was defined as a composite of ischemia-driven target lesion revascularization, target vessel-related myocardial infarction (MI), or cardiac death at 1 year. In this longer-term analysis from AGENT IDE, patients with vs without diabetes were compared using time-to-first event and repeat events methods through 2 years.

**Results:**

Among the 598 patients with known diabetes status, 51% had diabetes. At 2 years, rates of target vessel-related MI were higher among patients with vs without diabetes. There were no significant differences in 2-year TLF with paclitaxel-coated vs uncoated balloon angioplasty in patients with ISR and diabetes (29.3% vs 35.9%; hazard ratio, 0.78; 95% CI, 0.51-1.19) or those without diabetes (24.2% vs. 32.2%; hazard ratio, 0.66; 95% CI, 0.42-1.04) (*P*_i__nt_ = .62). Paclitaxel-coated balloon angioplasty was associated with a trend toward lower rates of ischemia-driven target lesion revascularization regardless of diabetes status. There were no instances of stent thrombosis in patients undergoing paclitaxel-coated balloon angioplasty.

**Conclusions:**

Among patients undergoing percutaneous coronary intervention for ISR, the presence vs absence of diabetes was associated with higher rates of total target vessel-related MI and TLF. Diabetes did not appear to modify the treatment effect of paclitaxel-coated vs uncoated balloon angioplasty on TLF, with point estimates favoring treatment with paclitaxel-coated balloons over uncoated balloon angioplasty in both groups. However, these subgroup findings were underpowered.

## Introduction

Diabetes is associated with a high risk of coronary in-stent restenosis (ISR) in patients undergoing percutaneous coronary intervention (PCI), often resulting in major adverse cardiovascular events and additional revascularization procedures.^1^^,^^2^ This association has been attributed to the diffuse pattern of disease, negative vessel remodeling, and metabolic changes that result in accelerated intimal hyperplasia, increased vascular inflammation, and endothelial dysfunction in patients with diabetes.^2^^,^^3^ In order to treat these mechanisms of coronary restenosis, drug-coated balloons have emerged as a potential therapy. The treatment of coronary ISR in the United States has traditionally consisted of uncoated balloon angioplasty followed by the placement of drug-eluting stents to restore coronary blood flow. In March 2024, the US Food and Drug Administration approved the AGENT paclitaxel-coated balloon for the treatment of coronary ISR as a result of the reduction in target lesion failure (defined as a composite of ischemia-driven target lesion revascularization, target vessel-related myocardial infarction [MI], and cardiac death) at 1 year with paclitaxel-coated vs uncoated balloon angioplasty.^4^ Whether the efficacy and safety of the AGENT paclitaxel-coated balloon angioplasty observed in the original trial is consistent in the higher risk cohort of patients with diabetes remains unclear.

As such, the objective of the present study was to understand the efficacy and safety of the paclitaxel-coated vs uncoated balloon angioplasty in patients with vs without diabetes and coronary ISR.

## Methods

### Study design and patient population

AGENT IDE (A Clinical Trial to Assess the Agent Paclitaxel-Coated Percutaneous Transluminal Coronary Angioplasty Balloon Catheter for the Treatment of Subjects With In-Stent Restenosis) was a multicenter, single-blind, randomized controlled, superiority trial that enrolled 600 patients with coronary IRS across 40 sites in the United States (ClinicalTrials.gov; NCT04647253).^4^ The study compared angioplasty with the AGENT paclitaxel-coated balloon vs an uncoated balloon in a 2:1 fashion. Key inclusion criteria included a reference vessel diameter >2.0 mm and up to 4.0 mm, lesion length <26 mm, and target lesion stenosis of >50% for symptomatic or >70% for asymptomatic patients. Dual antiplatelet therapy (DAPT), consisting of aspirin and a P2Y12 inhibitor, was prescribed for at least 1 month following the procedure, after which antiplatelet monotherapy was initiated and maintained throughout the study period. All subjects provided informed consent to participate in the trial. Institutional review board approvals were obtained from all relevant institutions.

In this prespecified subgroup analysis of AGENT IDE, patients were evaluated based on the presence or absence of diabetes at baseline. Patients were categorized as having diabetes if medical treatment (oral or injection) was required for control of blood glucose levels at baseline. The trial was performed in accordance with the principles of the Declaration of Helsinki, and the study design adheres to the CONSORT 2010 Statement for randomized clinical trials. The first author wrote the first draft of the manuscript, and all coauthors revised and reviewed it prior to submission.

### Clinical end points

Patient follow-up occurred at 30 days, 6 months, 12 months, and 24 months. As in the original trial, the primary end point for this analysis was target lesion failure, defined as a composite of ischemia-driven target lesion revascularization, target vessel-related MI, and cardiac death. Given the extended follow-up available, outcomes were reported through 2 years. Secondary end points included individual components of target lesion failure—ischemia-driven target lesion revascularization, target vessel-related MI, and cardiac death—as well as target vessel revascularization either in target lesion or not in target lesion, target vessel failure (defined as the composite of ischemia-driven target vessel revascularization, MI related to the target vessel, or cardiac death), all death, noncardiac death, and stent thrombosis as defined by the Academic Research Consortium^5^ at 2 years.

### Statistical analysis

Continuous variables are presented as mean ± SD, and differences between groups were compared using 2-sided *t* tests. Discrete variables are reported as percentages and counts, and differences were compared using the chi-square or Fisher exact tests. In the primary analysis, time-to-first-event methods using the Kaplan-Meier survival analysis were employed to estimate cumulative rates through 1 and 2 years. Cox models were used to calculate hazard ratios (HR) and their corresponding 95% CIs. Group comparisons (diabetes vs no diabetes and paclitaxel-coated vs uncoated balloons) were conducted using log-rank and Wilcoxon tests. Interactions between the randomized treatment effect and diabetes status were evaluated using the Cox proportional hazard regression model.

Because patients with restenosis commonly have a recurrent nonfatal events,^6^ we additionally conducted a total events analysis, including first and recurrent events, by estimating the mean cumulative count for each group and comparing them using the Lin Wei Yang Ying (LWYY) model.^7^ Additional exploratory analyses were performed in patients with diabetes, on vs off insulin therapy, and in those with multilayer ISR.

All tests conducted were 2-sided, and *P* < .05 was considered statistically significant with no adjustment for multiple testing. Statistical analyses were performed using SAS version 9.4 (SAS Institute).

## Results

### Study population and baseline characteristics

**Figure 1:**
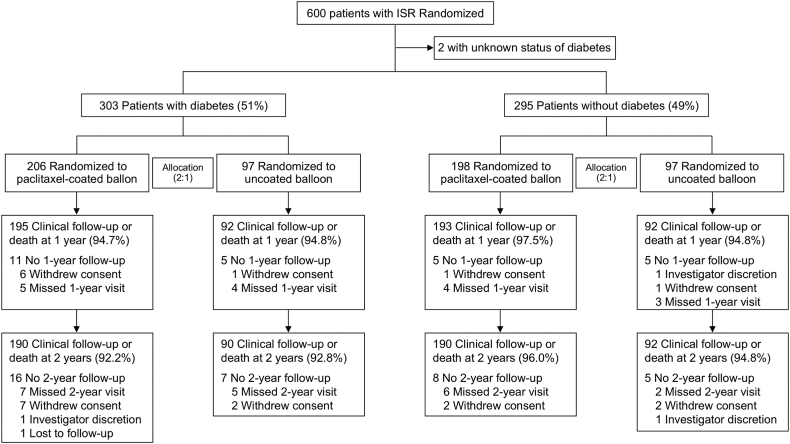
**Study CONSORT Diagram.** ISR, in-stent restenosis.

Among 598 patients with known diabetes status, 303 (51%) patients had diabetes. Allocation to either paclitaxel-coated or uncoated balloon angioplasty in patients with or without diabetes remained 2:1 as shown in the study consort diagram (Figure 1). Two-year follow-up was achieved in 94% of enrolled patients.

Baseline characteristics were largely well balanced between patients with vs without diabetes (Table 1). However, patients with diabetes were more likely to have greater body mass indexes (30.8 vs 29.3 kg/m^2^) and higher rates of congestive heart failure (27.0% vs 17.8%) and renal disease (23.2% vs 12.2%) compared with those without diabetes (*P* < .001 for all). The presence of multilayer ISR was similar between the 2 groups (44.2% vs 42.2%), as were lesion characteristics, including lesion length (12.7 mm vs 12.3 mm) and reference vessel diameter (2.7 mm vs 2.7 mm). Procedural and postprocedural outcomes by diabetes status are presented in Supplemental Table S1. Technical success rates were similar between patients with vs without diabetes. Use of intravascular imaging was upwards of 70% in both groups, and 1 patient with diabetes vs 3 without diabetes received bailout stenting.

**Table 1:** Baseline characteristics of patients with coronary ISR with or without diabetes

Characteristics	Diabetes (n = 303)	No diabetes (n = 295)	*P* value
Age, y	67.6 ± 9.1	69.0 ± 10.3	.08
Sex			
Male	73.6% (223/303)	73.9% (218/295)	.93
Female	26.4% (80/303)	26.1% (77/295)	.93
Race or ethnicity			
White	70.6% (214/303)	80.3% (237/295)	.006
Black	8.6% (26/303)	5.4% (16/295)	.13
Asian	2.6% (8/303)	2.4% (7/295)	.83
Hispanic	8.3% (25/303)	3.1% (9/295)	.006
American Indian or Alaska native	0.3% (1/303)	0% (0/295)	>.99^a^
Native Hawaiian or Pacific Islander	0.7% (2/303)	0% (0/295)	.50^a^
Other	3.3% (10/303)	3.1% (9/295)	.86
Not disclosed	7.6% (23/303)	6.1% (18/295)	.47
Clinical factors			
Body mass index, kg/m^2^	30.8 ± 5.7 (n = 294)	29.3 ± 5.4 (n = 291)	<.001
Currently smokes	9.9% (30/303)	10.5% (31/295)	.81
Hypertension requiring medication	96.4% (292/303)	93.5% (275/294)	.11
Hyperlipidemia requiring medication	96.0% (290/302)	93.2% (274/294)	.13
Multivessel coronary artery disease	81.3% (244/300)	76.4% (220/288)	.14
Prior myocardial infarction	52.7% (157/298)	46.5% (134/288)	.14
Prior CABG	32.4% (97/299)	27.6% (81/294)	.19
Congestive heart failure	27.0% (81/300)	17.8% (52/292)	<.001
Left main coronary artery disease	22.2% (66/297)	21.6% (61/283)	.85
Peripheral vascular disease	18.8% (56/298)	17.7% (52/293)	.74
Renal disease	23.2% (69/298)	12.2% (36/295)	<.001
Transient ischemic attack or cerebrovascular accident	13.6% (41/301)	11.3% (33/293)	.38
Lesion characteristics			
Stent layer in target lesion			
Single	55.8% (169/303)	57.8% (170/294)	.61
Multiple	44.2% (134/303)	42.2% (124/294)	.61
Target lesion vessel			
Right coronary artery	32.7% (99/303)	42.7% (126/295)	.01
Left anterior descending artery	34.0% (103/303)	35.9% (106/295)	.62
Left circumflex artery	29.0% (88/303)	19.0% (56/295)	.004
Left main coronary artery	4.3% (13/303)	2.4% (7/295)	.19
Lesion length, mm	12.7 ± 6.4 (299)	12.3 ± 6.4 (291)	.40
Reference vessel diameter, mm	2.7 ± 0.5 (302)	2.7 ± 0.5 (293)	.09
In-lesion minimum lumen diameter, mm	0.9 ± 0.4 (301)	1.0 ± 0.4 (293)	.01
In-lesion diameter stenosis, %	66.4 ± 12.6 (301)	64.5 ± 11.9 (293)	.06

Data are presented as mean ± SD or % (n/N) unless otherwise noted.

*P* values are 2-sided. For categorical variables, *P* values were calculated using either the *χ*^2^ test or Fisher exact test; results from Fisher exact test are denoted with a superscript letter (^a^). For continuous variables, *P* values were calculated using the *t* test.

Most patients continued DAPT through 24 months irrespective of diabetes status (Supplemental Table S2). Among patients with diabetes, DAPT was used in 69.7% of patients treated with the paclitaxel-coated balloon vs 78.8% of those treated with uncoated balloon angioplasty at 2 years of follow-up, whereas in those without diabetes, DAPT was used in 65.5% of patients in either treatment arm.

### Clinical outcomes

#### Diabetes vs no diabetes

At 2 years, the cumulative incidence of target vessel-related MI (time-to-first event) was higher in patients with vs without diabetes (12.7% vs 5.9%; *P* = .005) (Table 2) as were total target vessel-related MI events, including both first and recurrent events (0.14 vs 0.07; HR, 2.14; 95% CI, 1.13-4.03; *P* = .02) (Table 3). Similarly, total target lesion failure events were also higher at 0.53 in patients with diabetes vs 0.38 in those without diabetes (HR, 1.38; 95% CI, 1.01-1.89; *P* = .04) (Table 3). There were no statistically significant differences between rates of ischemia-driven target vessel revascularization or cardiac death by diabetes status. Instances of definite or probable stent thrombosis were low in both groups (1.4% vs 0.7%; HR, 1.98; 95% CI, 0.36-10.78; *P* = .42) (Table 2).

**Table 2:** Two-year clinical outcomes (time-to-first event analysis) after revascularization according to the presence or absence of diabetes in patients from AGENT IDE

Outcomes	Diabetes (n = 303)	No diabetes (n = 295)	Hazard ratio (95% CI)	*P* value
Target lesion failure	31.4% (93)	26.8% (77)	1.26 (0.93-1.70)	.14
Target lesion revascularization				
Overall	24.2% (70)	22.4% (63)	1.14 (0.81-1.61)	.44
PCI	20.3% (59)	17.4% (49)	1.24 (0.85-1.81)	.27
CABG	5.8% (16)	6.4% (18)	0.89 (0.45-1.74)	.73
Target vessel revascularization				
Overall	27.1% (78)	23.8% (67)	1.20 (0.87-1.67)	.27
PCI	23.1% (67)	18.9% (53)	1.30 (0.91-1.87)	.15
CABG	6.1% (17)	6.4% (18)	0.94 (0.49-1.83)	.87
Target vessel failure	33.9% (100)	27.9% (80)	1.31 (0.97- 1.75)	.07
Myocardial infarction	15.9% (46)	6.6% (19)	2.49 (1.46-4.26)	<.001
Related to target vessel	12.7% (37)	5.9% (17)	2.23 (1.26-3.96)	.005
Q-wave MI	0.7% (2)	0% (0)	—	.16
Related to target vessel	0.3% (1)	0% (0)	—	.32
Non-Q-wave MI	15.2% (44)	6.6% (19)	2.37 (1.39-4.07)	.001
Related to target vessel	12.4% (36)	5.9% (17)	2.16 (1.22-3.85)	.007
Not related to target vessel	3.6% (10)	0.7% (2)	5.03 (1.10-22.95)	.02
All death	6.9% (20)	7.8% (22)	0.91 (0.50-1.68)	.77
Cardiac	4.5% (13)	3.9% (11)	1.18 (0.53-2.63)	.69
Noncardiac	2.5% (7)	4.0% (11)	0.65 (0.25-1.67)	.36
Definite or probable stent thrombosis	1.4% (4)	0.7% (2)	1.98 (0.36-10.78)	.42

For all end points, percentage and 95% CI values indicate Kaplan-Meier estimates.

Data are presented as n (%), unless otherwise noted.

CABG, coronary artery bypass graft surgery; MI, myocardial infarction; PCI, percutaneous coronary intervention.

**Table 3:** Total (first and recurrent) target lesion failure events (mean cumulative count per patient) through 2 years according to the presence or absence of diabetes in patients from AGENT IDE

Events	Mean cumulative count		Hazard ratio (95% CI)	*P* value
	Diabetes n = 303	No diabetes n = 295		
Target lesion failure	0.56	0.41	1.38 (1.01-1.89)	.04
Target lesion revascularization	0.36	0.29	1.22 (0.86-1.71)	.27
Target vessel-related myocardial infarction	0.15	0.08	2.14 (1.13-4.03)	.02
Cardiac death	0.05	0.04	1.18 (0.53-2.62)	.69

Analyses were performed using LWYY model. Cumulative mean event counts through 2 years were reported.

#### Paclitaxel-coated vs uncoated balloon angioplasty

**Figure 2:**
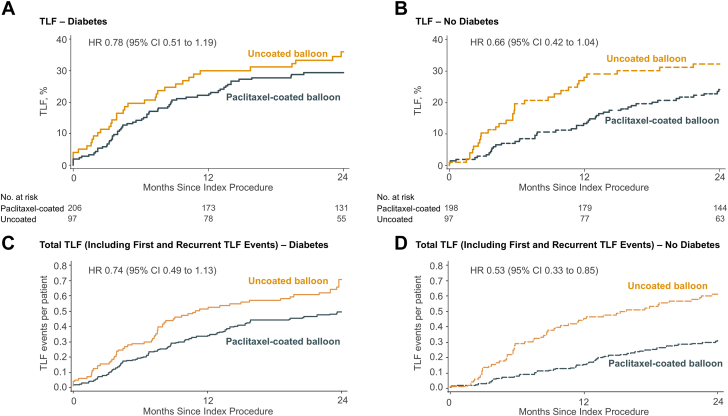
**First and total target lesion failure events through 2 years by diabetes status and angioplasty method.** Cumulative incidence of first (**A** and **B**) and total (**C** and **D**) target lesion failure events (mean cumulative count per patient) through 2 years in patients with coronary ISR with versus without diabetes undergoing paclitaxel-coated (navy) vs uncoated (gold) balloon angioplasty.

**Figure 3:**
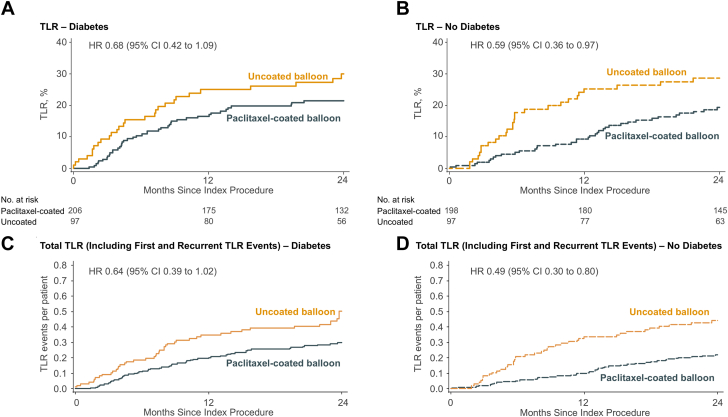
**First and total ischemia-driven target lesion revascularization events through 2 years by diabetes status and angioplasty method.** Cumulative incidence of first (**A** and **B**) and total (**C** and **D**) target lesion revascularization events (mean cumulative count per patient) through 2 years in patients with coronary ISR with versus without diabetes undergoing paclitaxel-coated (navy) vs uncoated (gold) balloon angioplasty.

Kaplan-Meier estimates of time-to-first clinical events by diabetes status and randomized treatment are shown through 1 year in Supplemental Table S3 and through 2 years in Table 4. Target lesion failure through 2 years occurred in 29.3% of patients with diabetes after paclitaxel-coated balloon angioplasty compared with 35.9% after uncoated balloon angioplasty (HR, 0.78; 95% CI, 0.51-1.19) and in 24.2% vs 32.2% patients without diabetes (HR, 0.66; 95% CI, 0.42-1.04) (*P*_int_ = .62) (Figure 2A, B, Central Illustration). Paclitaxel-coated balloon angioplasty was associated with a trend toward lower ischemia-driven target lesion revascularization compared with uncoated balloon angioplasty (Figure 3A, B). No significant differences were noted by treatment method or diabetes status in the 2-year rates of target vessel failure, target vessel-related MI, all-cause, cardiac, or noncardiac death.

**Table 4:** Two-year clinical outcomes (time-to-first event analysis) after paclitaxel-coated vs uncoated balloon angioplasty according to the presence or absence of diabetes in patients from AGENT IDE.

	Diabetes				No diabetes				*P* for interaction
	Paclitaxel-coated balloon n = 206	Uncoated balloon n = 97	Hazard ratio (95% CI)	*P* value	Paclitaxel-coated balloon n = 198	Uncoated balloon n = 97	Hazard ratio (95% CI)	*P* value	
Target lesion failure	29.3% (59)	35.9% (34)	0.78 (0.51-1.19)	.25	24.2% (46)	32.2% (31)	0.66 (0.42-1.04)	.07	.62
Target lesion revascularization									
Overall	21.5% (42)	30.0% (28)	0.68 (0.42-1.09)	.11	19.3% (36)	28.6% (27)	0.59 (0.36-0.97)	.04	.72
PCI	16.3% (32)	28.4% (27)	0.53 (0.32-0.88)	.01	14.1% (26)	24.3% (23)	0.50 (0.29-0.88)	.01	.94
CABG	6.2% (12)	4.8% (4)	1.45 (0.47-4.50)	.52	5.3% (10)	8.5% (8)	0.60 (0.24-1.52)	.28	.24
Target vessel revascularization									
Overall	24.1% (47)	33.4% (31)	0.68 (0.43-1.08)	.098	21.0% (39)	29.6% (28)	0.61 (0.38-0.99)	.04	.76
PCI	19.0% (37)	31.8% (30)	0.55 (0.34-0.88)	.01	15.8% (29)	25.3% (24)	0.53 (0.31-0.92)	.02	.97
CABG	6.7% (13)	4.8% (4)	1.58 (0.51-4.84)	.42	5.3% (10)	8.5% (8)	0.60 (0.24-1.52)	.28	.19
Target vessel failure	31.4% (63)	39.2% (37)	0.76 (0.51-1.14)	.19	25.4% (48)	33.3% (32)	0.66 (0.42-1.04)	.07	.67
Myocardial infarction (MI)	14.9% (29)	17.9% (17)	0.78 (0.43-1.42)	.42	5.2% (10)	9.5% (9)	0.53 (0.22-1.30)	.16	.47
Related to target vessel	11.2% (22)	15.7% (15)	0.67 (0.35-1.30)	.24	4.7% (9)	8.5% (8)	0.54 (0.21-1.39)	.19	.70
Q-wave MI	0.5% (1)	1.0% (1)	0.47 (0.03-7.54)	.59	0% (0)	0% (0)	—	Undef	>.99
Related to target vessel	0% (0)	1.0% (1)	—	.15	0% (0)	0% (0)	—	Undef	>.99
Non-Q-wave MI	14.4% (28)	16.9% (16)	0.81 (0.44-1.49)	.50	5.2% (10)	9.5% (9)	0.53 (0.22-1.30)	.16	.44
Related to target vessel	11.2% (22)	14.7% (14)	0.73 (0.37-1.43)	.36	4.7% (9)	8.5% (8)	0.54 (0.21-1.39)	.19	.61
Not related to target vessel	3.8% (7)	2.2% (2)	1.67 (0.35-8.03)	.52	0.5% (1)	1.0% (1)	0.49 (0.03-7.79)	.60	.45
	7.6% (15)	5.3% (5)	1.44 (0.52-3.95)	.48	7.6% (14)	8.4% (8)	0.86 (0.36-2.05)	.73	.45
Cardiac	5.6% (11)	2.2% (2)	2.64 (0.58-11.90)	.19	3.8% (7)	4.3% (4)	0.86 (0.25-2.93)	.81	.26
Noncardiac	2.2% (4)	3.2% (3)	0.64 (0.14-2.84)	.55	3.9% (7)	4.4% (4)	0.86 (0.25-2.94)	.81	.76
Definite or probable stent thrombosis	0% (0)	4.2% (4)	—	.004	0% (0)	2.1% (2)	—	.04	>.99

For all end points, percentage and 95% CI values indicate Kaplan-Meier estimates.

Data are presented as n (%) or n/N (%) (when N differs from column N due to missing values), unless otherwise noted.

CABG, coronary artery bypass graft surgery; MI, myocardial infarction; PCI, percutaneous coronary intervention; Undef, undefined.

**Central Illustration:**
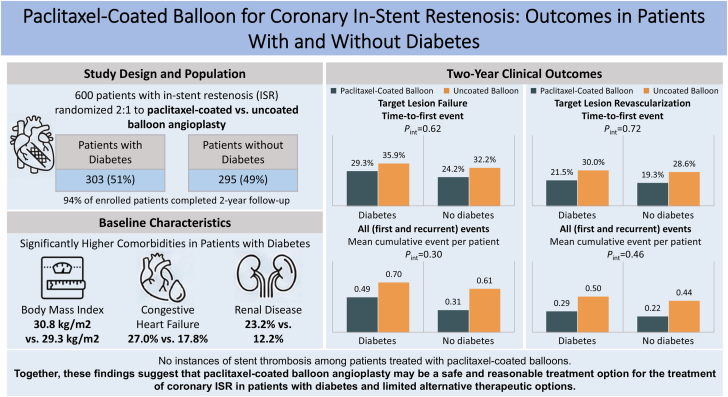
**Paclitaxel-coated balloon for in-stent restenosis: outcomes in patients with and without diabetes.** Among the 600 patients with ISR randomized in a 2:1 fashion in AGENT IDE, 51% had diabetes. Patients with diabetes had higher comorbidities, including higher body mass index, congestive heart failure, and renal disease. Overall, diabetes did not modify the treatment effect of paclitaxel-coated balloon angioplasty on the primary outcome of target lesion failure. However, there was a consistent trend toward lower target lesion revascularization with paclitaxel-coated balloons in patients with and without diabetes. Altogether, these findings suggest that paclitaxel-coated balloon angioplasty may be a safe and reasonable treatment option for the treatment of coronary ISR in patients with diabetes and limited alternative therapies.

In the total (first and recurrent) events analysis, among patients with diabetes, the mean cumulative count of target lesion failure events was 0.49 after paclitaxel-coated balloon angioplasty vs 0.70 events after uncoated balloon angioplasty (HR, 0.74; 95% CI, 0.49-1.13), compared with 0.31 vs 0.61 target lesion failure events among those without diabetes (HR, 0.53; 95% CI, 0.33-0.85) (*P*_int_ = .30) (Figure 2C, D, Table 5). The numerical reduction in total target lesion failure events with paclitaxel-coated balloon angioplasty was driven principally by reductions in target lesion revascularization that trended toward significance in patients with diabetes (HR, 0.64; 95% CI, 0.39-1.02) and were significant in those without diabetes (HR, 0.49; 95% CI, 0.30-0.80) (*P*_int_ = .46) (Figure 3C, D, Table 5).

**Table 5:** Total end point events (including first and recurrent events) on a per patient basis through 2 years after paclitaxel-coated vs uncoated balloon angioplasty according to the presence or absence of diabetes in patients from AGENT IDE

	Diabetes				No diabetes				*P* for Interaction
	Mean cumulative count		Hazard ratio (95% CI)	*P* value	Mean cumulative count		Hazard ratio (95% CI)	*P* value	
	Paclitaxel-coated balloon N = 206	Uncoated balloon N = 97			Paclitaxel-coated balloon N = 198	Uncoated balloon N = 97			
Target lesion failure	0.49	0.70	0.74 (0.49-1.13)	.16	0.31	0.61	0.53 (0.33-0.85)	.008	.30
Target lesion revascularization	0.29	0.50	0.64 (0.39-1.02)	.06	0.22	0.44	0.49 (0.30-0.80)	.005	.46
Target vessel-related myocardial infarction	0.14	0.18	0.68 (0.35-1.31)	.24	0.05	0.13	0.36 (0.12-1.04)	.06	.32
Cardiac death	0.06	0.02	2.64 (0.59-11.79)	.20	0.04	0.04	0.86 (0.25-2.93)	.81	.26

Analyses were performed using the LWYY model. Cumulative mean event counts through 2 years were reported.

Incident rates of stent thrombosis are displayed in Table 4. There were no instances of definite or probable stent thrombosis among patients treated with paclitaxel-coated balloon angioplasty, irrespective of diabetes status, up to 2 years of follow-up. Among those treated with uncoated balloon angioplasty, 4 patients with diabetes and 2 without diabetes experienced definite or probable stent thrombosis.

When patients with diabetes were further stratified by insulin therapy, there were no significant interactions between insulin therapy status and ISR treatment strategy (Supplemental Table S4).

Since more than 40% of patients had multilayer ISR, exploratory analyses comparing treatment strategies were performed in this subpopulation. In patients with diabetes, paclitaxel-coated balloon angioplasty was associated with a significant reduction in target lesion failure, target vessel-related MI, and ischemia-driven target lesion revascularization when compared with uncoated balloon angioplasty (Supplemental Table S5). In those without diabetes, there was no statistically significant difference with respect to any of the outcomes with paclitaxel-coated vs uncoated balloon angioplasty.

## Discussion

In this prespecified analysis comparing patients with vs without diabetes from the AGENT IDE trial, patients with diabetes had a significantly higher risk of total target lesion failure and target vessel-related MI events compared with those without diabetes. When stratified by ISR treatment strategy, there were no significant differences in the rates of target lesion failure with paclitaxel-coated vs uncoated balloon angioplasty, irrespective of diabetes status. However, paclitaxel-coated balloon angioplasty was associated with reductions in ischemia-driven target lesion revascularization compared with uncoated balloon angioplasty. These findings were more pronounced when accounting for all events that occurred over the study period and in patients with multilayer ISR and diabetes. There were no instances of stent thrombosis among patients treated with the paclitaxel-coated balloon, irrespective of diabetes status at up to 2 years of follow-up.

Diabetes is associated with an elevated risk of coronary restenosis after PCI. According to the 2023 SCAI Expert Consensus Statement, rates of ISR after PCI with second-generation drug-eluting stents are 5.7% in patients without diabetes and significantly higher at 8.7% in those with diabetes.^8^ Other studies have noted similarly elevated rates of restenosis among patients with diabetes.^9^^,^^10^ Mechanistically, increased rates of ISR with diabetes have been attributed to accelerated intimal hyperplasia as well as heightened vascular inflammation and endothelial dysfunction.^2^ In addition, patients with diabetes have been shown to have long and diffuse coronary lesions when compared with those without diabetes, which in itself may contribute to a higher risk of restenosis.^3^ Given this increased mechanistic risk, it is feasible that angioplasty with local administration of antiproliferative therapy via a drug-coated balloon may result in lower rates of localized neointimal hyperplasia and restenosis in this higher risk population.

The AGENT drug-coated balloon is a semicompliant coronary balloon with a low-dose formation of paclitaxel, an antiproliferative drug, and the excipient acetyl tri-n-butyl citrate that allows the drug to be transferred to the vessel wall and dwell for an average of 90 days.^4^ AGENT IDE, the first trial of coronary drug-coated balloons performed in the United States, demonstrated a reduction in target lesion failure with paclitaxel-coated vs uncoated balloon angioplasty up to 1 year after randomization. Recent follow-up of the trial confirmed the ongoing efficacy and safety of the paclitaxel-coated balloons up to 2 years. However, few studies have previously evaluated the relationship between diabetes status and clinical outcomes after drug-coated balloon angioplasty for coronary ISR.

In the present study, there were no statistical differences in the rates of target lesion failure with paclitaxel-coated vs uncoated balloon angioplasty by diabetes status over 2 years of follow-up. Although the presence or absence of diabetes did not significantly impact the primary end point, paclitaxel-coated angioplasty was associated with a trend toward lower rates of ischemia-driven target lesion revascularization, consistent with the overall trial results. To improve the statistical power of the analyses, we performed total event analyses, which remained directionally consistent and revealed a lower risk of repeat events in patients undergoing paclitaxel vs uncoated balloon angioplasty. There were no significant differences by treatment strategy with respect to rates of target vessel failure, target vessel MI, all-cause, cardiac, or noncardiac death, regardless of diabetes status.

Considering the antiproliferative effect of paclitaxel-coated balloons, one might have expected a more robust reduction in these end points with drug-coated balloon vs uncoated balloon treatment, particularly in patients with diabetes, but we did not observe such a difference. The presence of diabetes did not seem to modify the treatment effect in the lesions studied in AGENT IDE. There are several potential explanations for these findings, including the similar baseline lesion characteristics among patients with vs without diabetes enrolled in AGENT IDE, including similar lesion lengths (12.7 mm vs 12.3 mm) and reference vessel diameter (2.7 mm vs 2.7 mm). As such, it remains unknown whether paclitaxel-coated balloon angioplasty would perform better in longer lesions with more ISR that are more characteristic in patients with diabetes.^11^ In addition, most patients continued DAPT through 24 months irrespective of diabetes status. Prolonged DAPT use may have attenuated differences in thrombotic outcomes observed between the treatment strategies. While the high and relatively balanced use of prolonged DAPT across treatment arms suggests against such a differential effect, residual confounding cannot be excluded, and thus the current findings should be interpreted within this context.

When the subset of patients with multilayer ISR was analyzed, in those with diabetes, there was a significant reduction in the rates of target lesion failure, target vessel-related MI, and target lesion revascularization with paclitaxel-coated vs uncoated balloon angioplasty. This represents an important area for further investigation.

There were 6 cases of definite or probable stent thrombosis in patients treated with uncoated balloon angioplasty, including 4 patients with diabetes and 2 without diabetes. Reassuringly, there were no instances of stent thrombosis in patients treated with the paclitaxel-coated balloon, highlighting the ongoing safety of drug-coated balloons. The safety of drug-coated balloons has been further supported by data from a patient-level meta-analysis of 10 randomized clinical trials comparing drug-coated balloons with drug-eluting stents, which showed no increase in safety adverse events, including restenosis, among those treated with paclitaxel-coated balloons.^12^

In Europe, the ESC/EACTS guideline committees have endorsed the use of drug-coated balloons for the treatment of ISR since the 2018 guideline update.^13^ However, the specific advantage of this therapy in patients with diabetes and ISR has received less emphasis. In the United States, the 2021 SCAI/ACC/AHA guidelines on coronary revascularization omit any mention of drug-coated balloons for the treatment of patients with stent restenosis. The 2023 SCAI Expert Consensus Statement does acknowledge diabetes as a contributory risk factor for ISR but does not yet explicitly recommend the use of paclitaxel-coated balloons as a treatment strategy in this population. Given the limited therapeutic options available to patients with coronary ISR, particularly those with multilayer ISR, together with the aforementioned study findings, we believe that paclitaxel-coated balloon angioplasty is a safe and reasonable treatment alternative for patients with diabetes who develop coronary ISR.

### Limitations

We acknowledge several limitations to this analysis. First, all patients enrolled in AGENT IDE had ISR with a reference vessel diameter of >2 mm and up to 4 mm and a length <26 mm with target lesion stenosis of <100% but >50% (symptomatic) and >70% (asymptomatic). Moreover, intravascular imaging utilization was greater than 70% irrespective of diabetes status, and a majority of patients continued on DAPT through 2 years. This may limit the generalizability of our findings to conventional clinical practice, especially in geographies where imaging is less frequently performed or for patients in whom shorter durations of DAPT are used.^14^^,^^15^ Second, patients with diabetes had a higher prevalence of comorbid conditions, including congestive heart failure and renal disease. Residual confounding cannot be excluded. Third, AGENT IDE was not designed to compare drug-coated balloons to drug-eluting stents, which are the current standard treatment of ISR, and as a result, the findings should be interpreted in the appropriate context. Fourth, enrollment of patients in this trial predated the routine use of novel diabetes pharmacotherapies that have been shown to impact cardiovascular outcomes. In addition, there were per-protocol crossovers in the first year that may have attenuated the relative efficacy of the AGENT balloon. Finally, although this was a prespecified analysis of AGENT IDE, the study was not powered to specifically evaluate outcomes in patients with vs without diabetes. Thus, the findings are hypothesis generating.

## Conclusions

In this prespecified analysis of patients with coronary ISR undergoing PCI in AGENT IDE, the presence vs absence of diabetes was associated with a significantly higher risk of total target lesion failure and target vessel-related MI events. Diabetes did not appear to modify the treatment effect of paclitaxel-coated vs uncoated balloon angioplasty on target lesion failure. Nonetheless, the point estimates for the treatment effect favored paclitaxel-coated balloon angioplasty with regard to ischemia-driven target lesion revascularization in patients with and without diabetes. These findings were more pronounced when accounting for recurrent events and are consistent with the overall trial results. However, the study was not powered for this subgroup analysis, and subgroup treatment effects did not meet statistical significance. Taken together, these findings support the safety profile of paclitaxel-coated balloon angioplasty and suggest that it may be a viable treatment option for coronary ISR in patients with diabetes and limited alternative therapies.

## Declaration of competing interest

Brian Jefferson is a speaker and on the advisory board for Boston Scientific, a speaker for Shockwave Medical, and on the advisory board for Medtronic. Richard Shlofmitz reports speaker honoraria from Shockwave Medical. Jeffrey W. Moses reports equity in Orchestra BioMed. Suhail Dohad reports research grants consulting fees and speaker for Penumbra, Boston Scientific, Abbott Vascular, and Johnson & Johnson. Robert Stoler is part of the advisory board and proctor for Boston Scientific, Medtronic, and Biotronik. Robert Stoler serves on the medical advisory boards of Medtronic, Boston Scientific, Edwards Lifesciences, and Biotronik; provides consulting services to Medtronic, Boston Scientific, Edwards Lifesciences, and Biotronik; and serves as a proctor for Medtronic, Boston Scientific, and Edwards Lifesciences. William Nicholson reports medical advisory board participation, educational grants, proctoring, and consulting with Boston Scientific, Asahi Intecc, Medtronic, and Teleflex; research grants from Philips; medical advisory board roles with Rampart and Avail; and intellectual property with Teleflex. Cinthia Bateman reports participation on the medical advisory board for Boston Scientific. Rafael Cavalcante is a full-time employee with equity interest in Boston Scientific. Ajay J. Kirtane reports institutional funding to Columbia University and/or the Cardiovascular Research Foundation from Medtronic, Boston Scientific, Abbott Vascular, Amgen, CathWorks, Concept Medical, Philips, Recor Medical, Neurotronic, Biotronik, Chiesi, Bolt Medical, Magenta Medical, SoniVie, and Shockwave Medical. In addition to research grants, Ajay J. Kirtane reports institutional funding includes fees paid to Columbia University and/or Cardiovascular Research Foundation for consulting and/or speaking engagements in which he controlled the content personal equity options in Bolt Medical and Airiver; and travel expenses/meals from Amgen, Medtronic, Biotronik, Boston Scientific, Abbott Vascular, CathWorks, Concept Medical, Novartis, Philips, Abiomed, Recor Medical, Chiesi, Zoll, Shockwave Medical, and Regeneron. Robert W. Yeh reports the following disclosures: research grants from Abbott Vascular, Boston Scientific, Edwards Lifesciences, Elixir Medical, JenaValve, Medtronic, and Siemens; consulting for Abbott Vascular, Boston Scientific, CathWorks, the Centers for Medicare and Medicaid Services, Edwards Lifesciences, Elixir Medical, JenaValve, Magenta Medical, and Medtronic; and employment as a Special Government Employee with the US Food and Drug Administration. The other authors reported no financial interests.

## References

[1] Kornowski R., Mintz G.S., Kent K.M. (1997). Increased restenosis in diabetes mellitus after coronary interventions is due to exaggerated intimal hyperplasia. Circulation.

[2] Konigstein M., Ben-Yehuda O., Smits P.C. (2018). Outcomes among diabetic patients undergoing percutaneous coronary intervention with contemporary drug-eluting stents: analysis from the BIONICS randomized trial. JACC Cardiovasc Interv.

[3] Aronson D., Edelman E.R. (2014). Coronary artery disease and diabetes mellitus. Cardiol Clin.

[4] Yeh R.W., Shlofmitz R., Moses J. (2024). Paclitaxel-coated balloon vs uncoated balloon for coronary in-stent restenosis: the AGENT IDE randomized clinical trial. JAMA.

[5] Cutlip D.E., Windecker S., Mehran R. (2007). Clinical end points in coronary stent trials: a case for standardized definitions. Circulation.

[6] Gregson J., Stone G.W., Bhatt D.L. (2023). Recurrent events in cardiovascular trials. J Am Coll Cardiol.

[7] Lin D.Y., Wei L.J., Yang I., Ying Z. (2000). Semiparametric regression for the mean and rate functions of recurrent events. J R Stat Soc B Stat Methodol.

[8] Klein L.W., Nathan S., Maehara A. (2023). SCAI Expert Consensus Statement on Management of In-Stent Restenosis and Stent Thrombosis. J Soc Cardiovasc Angiogr Interv.

[9] Paramasivam G., Devasia T., Jayaram A. (2020). In-stent restenosis of drug-eluting stents in patients with diabetes mellitus: clinical presentation, angiographic features, and outcomes. Anatol J Cardiol.

[10] Zhao L., Zhu W., Zhang X., He D., Guo C. (2017). Effect of diabetes mellitus on long-term outcomes after repeat drug-eluting stent implantation for in-stent restenosis. BMC Cardiovasc Disord.

[11] Cosgrave J., Melzi G., Biondi-Zoccai G.G. (2006). Drug-eluting stent restenosis the pattern predicts the outcome. J Am Coll Cardiol.

[12] Giacoppo D., Alfonso F., Xu B. (2020). Drug-coated balloon angioplasty versus drug-eluting stent implantation in patients with coronary stent restenosis. J Am Coll Cardiol.

[13] Neumann F.J., Sousa-Uva M., Ahlsson A. (2019). 2018 ESC/EACTS Guidelines on myocardial revascularization. Eur Heart J.

[14] Vrints C., Andreotti F., Koskinas K.C. (2024). 2024 ESC Guidelines for the management of chronic coronary syndromes. Eur Heart J.

[15] Truesdell A.G., Alasnag M.A., Kaul P. (2023). Intravascular imaging during percutaneous coronary intervention. J Am Coll Cardiol.

