# Supplementary material for: Efficacy and Safety of Paclitaxel-Coated Balloon Angioplasty in Patients With In-Stent Restenosis With vs Without Diabetes
Source: J Soc Cardiovasc Angiogr Interv. 2026 Apr 9;5(5):105320. doi: 10.1016/j.jscai.2026.105320 (PMC13198102; doi:10.1016/j.jscai.2026.105320)
Supplement: Supplementary Material [file mmc1.docx]

**SUPPLEMENT**

**Supplemental Table 1. Procedural and postprocedural characteristics of patients with coronary ISR with or without diabetes**

|  | Diabetes  (N = 303 subjects/304 lesions) | No Diabetes  (N = 295 subjects/lesions) | *P* value |
| --- | --- | --- | --- |
| **Procedural Outcomes** | |  | |
| Clinical procedural success | 92.1% (279/303) | 89.8% (265/295) | 0.34 |
| Technical success^a^ | 93.8% (285/304) | 90.5% (267/295) | 0.14 |
| Procedure time, min | 54.5±27.4 (302) | 55.9±30.2 (291) | 0.54 |
| Patients with only target lesion treated | 86.1% (261/303) | 88.1% (260/295) | 0.47 |
| Patients with both target & non-target lesion treated | 13.9% (42/303) | 11.9% (35/295) | 0.47 |
| Intravascular imaging usage any time during procedure^a^ | 73.3% (222/303) | 74.6% (220/295) | 0.72 |
| Bailout stenting | 0.3% (1/304) | 1.0% (3/295) | NA |
| Ancillary device usage |  | | |
| Balloon angioplasty catheter | 87.2% (265/304) | 88.1% (260/295) | 0.72 |
| Cutting balloon | 20.4% (62/304) | 29.2% (86/295) | 0.01 |
| Scoring balloon | 18.1% (55/304) | 10.8% (32/295) | 0.01 |
| Drug eluting stent | 1.0% (3/304) | 0.7% (2/295) | 1.00* |
| Bare metal stent | 0.0% (0/304) | 0.0% (0/295) | Undef |
| **Postprocedural Outcomes** | |  | |
| Hospital length of stay^b^, days | 0.73±1.56 (303) | 0.53±0.83 (295) | 0.06 |
| In-lesion^c^ minimum lumen diameter, mm | 2.08±0.46 (298) | 2.14±0.46 (293) | 0.12 |
| In-lesion^c^ diameter stenosis, % | 22.83±10.65 (296) | 22.29±9.96 (293) | 0.52 |
| In-lesion^c^ acute gain^d^, mm | 1.18±0.48 (294) | 1.17±0.45 (291) | 0.80 |

Data are presented as mean ± standard deviation, % (n/N) (when N differs from column N due to missing values), unless otherwise noted.

*P* values are two-sided. For categorical variables, *P* values were calculated using either the χ² test or Fisher’s exact test; results from Fisher’s exact test are denoted with an asterisk (*). For continuous variables, p-values were calculated using the t-test.

^a^One patient in the paclitaxel-coated balloon group had two target lesions treated with paclitaxel-coated balloons that was counted as a protocol deviation.

^b^Site reported.

^c^In-lesion refers to treated segment.

^d^Acute Gain = Post-procedure MLD – Pre-procedure MLD.

Abbreviations: NA, not available; Undef, undefined.

**Supplemental Table 2. Antiplatelet medications through 2 years among patients with vs without diabetes in AGENT IDE**

| Medication | Diabetes | | | No Diabetes | | |
| --- | --- | --- | --- | --- | --- | --- |
|  | Paclitaxel-coated balloon  (N=206) | Uncoated balloon  (N=97) | *P* value | Paclitaxel-coated balloon  (N=198) | Uncoated balloon  (N=97) | *P* value |
| Aspirin |  | | |  | | |
| Discharge | 96.1% (198/206) | 96.9% (94/97) | 1.00* | 98.5% (195/198) | 93.8% (91/97) | 0.06* |
| 30 Days | 92.1% (186/202) | 92.6% (87/94) | 0.89 | 93.9% (184/196) | 90.6% (87/96) | 0.31 |
| 6 Months | 89.3% (176/197) | 87.4% (83/95) | 0.62 | 88.6% (171/193) | 89.5% (85/95) | 0.82 |
| 12 Months | 88.2% (164/186) | 87.5% (77/88) | 0.87 | 84.9% (157/185) | 86.4% (76/88) | 0.74 |
| 24 Months | 86.3% (151/175) | 87.1% (74/85) | 0.86 | 80.8% (143/177) | 84.5% (71/84) | 0.46 |
| Aspirin and one of Clopidogrel, Ticlopidine, Prasugrel or Ticagrelor |  | | |  | | |
| Discharge | 96.1% (198/206) | 96.9% (94/97) | 1.00* | 98.0% (194/198) | 92.8% (90/97) | 0.04* |
| 30 Days | 91.6% (185/202) | 91.5% (86/94) | 0.98 | 92.9% (182/196) | 88.5% (85/96) | 0.22 |
| 6 Months | 86.8% (171/197) | 82.1% (78/95) | 0.29 | 83.9% (162/193) | 85.3% (81/95) | 0.77 |
| 12 Months | 78.5% (146/186) | 80.7% (71/88) | 0.68 | 78.9% (146/185) | 76.1% (67/88) | 0.60 |
| 24 Months | 69.7% (122/175) | 78.8% (67/85) | 0.12 | 65.5% (116/177) | 65.5% (55/84) | 0.99 |

*P* values are two-sided and were calculated using either the χ² test or Fisher’s exact test; results from Fisher’s exact test are denoted with an asterisk (*).

**Supplemental Table 3. One-year clinical outcomes (time-to-first event analysis) after paclitaxel-coated vs uncoated balloon angioplasty according to the presence or absence of diabetes in patients from AGENT IDE**

| Clinical outcomes | Diabetes | | | | No Diabetes | | | | *P* for interaction |
| --- | --- | --- | --- | --- | --- | --- | --- | --- | --- |
|  | Paclitaxel-coated balloon  (N = 206) | Uncoated balloon  (N = 97) | Hazard ratio  (95% CI) | *P* value | Paclitaxel-coated balloon  (N = 198) | Uncoated balloon  (N = 97) | Hazard ratio  (95% CI) | *P* value |  |
| Target lesion failure | 22.2% (45) | 30.2% (29) | 0.70 (0.44, 1.11) | 0.13 | 13.7% (26) | 26.9% (25) | 0.47 (0.27, 0.82) | 0.006 | 0.30 |
| Target lesion revascularization |  | | | |  | | | |  |
| Overall | 16.6% (33) | 25.1% (24) | 0.62 (0.37, 1.05) | 0.07 | 9.5% (18) | 24.1% (22) | 0.37 (0.20, 0.69) | 0.001 | 0.22 |
| PCI | 13.6% (27) | 25.2% (24) | 0.50 (0.29, 0.87) | 0.01 | 6.4% (12) | 20.5% (19) | 0.29 (0.14, 0.59) | 0.0003 | 0.23 |
| CABG | 3.5% (7) | 2.1% (2) | 1.66 (0.34, 7.97) | 0.52 | 3.1% (6) | 6.8% (6) | 0.48 (0.16, 1.50) | 0.20 | 0.22 |
| Target vessel revascularization |  | | | |  | | | |  |
| Overall | 18.2% (36) | 26.2% (25) | 0.65 (0.39, 1.08) | 0.09 | 10.6% (20) | 26.1% (24) | 0.37 (0.21, 0.67) | 0.0007 | 0.17 |
| PCI | 15.1% (30) | 26.2% (25) | 0.54 (0.31, 0.91) | 0.02 | 7.4% (14) | 22.4% (21) | 0.30 (0.15, 0.59) | 0.0002 | 0.18 |
| CABG | 4.0% (8) | 2.1% (2) | 1.90 (0.40, 8.94) | 0.41 | 3.1% (6) | 6.8% (6) | 0.48 (0.16, 1.50) | 0.20 | 0.16 |
| Target vessel failure | 23.2% (47) | 31.2% (30) | 0.70 (0.44, 1.11) | 0.13 | 13.7% (26) | 28.9% (27) | 0.43 (0.25, 0.74) | 0.002 | 0.18 |
| Myocardial infarction (MI) | 11.3% (22) | 15.9% (15) | 0.67 (0.35, 1.28) | 0.22 | 3.7% (7) | 8.4% (8) | 0.42 (0.15, 1.15) | 0.08 | 0.44 |
| Related to target vessel | 8.4% (17) | 14.8% (14) | 0.56 (0.27, 1.13) | 0.10 | 3.2% (6) | 7.4% (7) | 0.41 (0.14, 1.22) | 0.10 | 0.65 |
| Q-wave MI | 0.5% (1) | 1.0% (1) | 0.47 (0.03, 7.50) | 0.58 | 0.0% (0) | 0.0% (0) | NA | Undef | 1.00 |
| Related to target vessel | 0.0% (0) | 1.0% (1) | 0.00 (NA, NA) | 0.15 | 0.0% (0) | 0.0% (0) | NA | Undef | 1.00 |
| Non-Q-wave MI | 10.8% (21) | 14.9% (14) | 0.69 (0.35, 1.35) | 0.27 | 3.7% (7) | 8.4% (8) | 0.42 (0.15, 1.15) | 0.08 | 0.42 |
| Related to target vessel | 8.4% (17) | 13.8% (13) | 0.60 (0.29, 1.24) | 0.17 | 3.2% (6) | 7.4% (7) | 0.41 (0.14, 1.22) | 0.10 | 0.57 |
| Not related to target vessel | 2.4% (4) | 1.1% (1) | 1.86 (0.21, 16.61) | 0.57 | 0.5% (1) | 1.0% (1) | 0.49 (0.03, 7.79) | 0.60 | 0.46 |
| All death | 4.5% (9) | 4.3% (4) | 1.06 (0.33, 3.43) | 0.93 | 3.8% (7) | 3.2% (3) | 1.15 (0.30, 4.43) | 0.84 | 0.93 |
| Cardiac | 3.5% (7) | 1.1% (1) | 3.28 (0.40, 26.66) | 0.24 | 2.3% (4) | 2.1% (2) | 0.98 (0.18, 5.37) | 0.99 | 0.38 |
| Noncardiac | 1.0% (2) | 3.3% (3) | 0.31 (0.05, 1.88) | 0.18 | 1.6% (3) | 1.1% (1) | 1.47 (0.15, 14.09) | 0.74 | 0.29 |
| Definite or probable stent thrombosis | 0.0% (0) | 4.3% (4) | - | 0.003 | 0.0% (0) | 2.1% (2) | - | 0.04 | 1.00 |

For all endpoints, percentage and (95% CI) values indicate Kaplan-Meier estimates.

Data are presented as % (n) unless otherwise noted.

Abbreviations: CABG, coronary artery bypass graft surgery; CI, confidence interval; MI, myocardial infarction; NA, not available; PCI, percutaneous coronary intervention; Undef, undefined.

**Supplemental Table 4. Two-year clinical outcomes (time-to-first event analysis) after paclitaxel-coated vs uncoated balloon angioplasty in patients with insulin-treated vs non-insulin treated diabetes**

|  | Insulin-Treated Diabetes | | | Non-Insulin Treated Diabetes | | | *P* for Interaction |
| --- | --- | --- | --- | --- | --- | --- | --- |
|  | Paclitaxel-coated balloon  (N=93) | Uncoated balloon  (N=49) | Hazard ratio  (95% CI) | Paclitaxel-coated balloon  (N=113) | Uncoated balloon  (N=48) | Hazard ratio  (95% CI) |  |
| **Target lesion failure** | 29.9% (27) | 45.9% (22) | 0.59 (0.34, 1.04) | 28.9% (32) | 25.5% (12) | 1.15 (0.59, 2.23) | 0.13 |
| **Target lesion revascularization** |  |  |  |  |  |  |  |
| Overall | 21.7% (19) | 37.8% (18) | 0.53 (0.28, 1.01) | 21.2% (23) | 21.6% (10) | 0.96 (0.46, 2.01) | 0.24 |
| PCI | 16.0% (14) | 35.0% (17) | 0.41 (0.20, 0.83) | 16.5% (18) | 21.6% (10) | 0.74 (0.34, 1.60) | 0.26 |
| CABG | 5.7% (5) | 9.2% (4) | 0.69 (0.19, 2.57) | 6.6% (7) | 0.0% (0) | NA | 0.99 |
| **Target vessel revascularization** |  |  |  |  |  |  |  |
| Overall | 24.1% (21) | 40.1% (19) | 0.55 (0.30, 1.03) | 24.0% (26) | 26.1% (12) | 0.90 (0.46, 1.79) | 0.30 |
| PCI | 18.4% (16) | 37.2% (18) | 0.44 (0.22, 0.86) | 19.3% (21) | 26.1% (12) | 0.72 (0.35, 1.46) | 0.31 |
| CABG | 5.7% (5) | 9.2% (4) | 0.69 (0.19, 2.57) | 7.4% (8) | 0.0% (0) | NA | 0.99 |
| **Target vessel failure** | 32.2% (29) | 48.1% (23) | 0.60 (0.35, 1.04) | 30.7% (34) | 29.8% (14) | 1.04 (0.56, 1.93) | 0.20 |
| **Myocardial infarction (MI)** | 18.2% (16) | 24.9% (12) | 0.66 (0.31, 1.40) | 12.3% (13) | 10.8% (5) | 1.11 (0.39, 3.10) | 0.42 |
| Related to target vessel | 13.6% (12) | 24.9% (12) | 0.50 (0.22, 1.11) | 9.4% (10) | 6.4% (3) | 1.43 (0.39, 5.20) | 0.17 |
| Q-wave MI | 1.1% (1) | 2.0% (1) | 0.53 (0.03, 8.43) | 0.0% (0) | 0.0% (0) | NA | 1.00 |
| Related to target vessel | 0.0% (0) | 2.0% (1) | 0.00 (NA, NA) | 0.0% (0) | 0.0% (0) | NA | 1.00 |
| Non-Q-wave MI | 17.1% (15) | 22.8% (11) | 0.69 (0.32, 1.51) | 12.3% (13) | 10.8% (5) | 1.11 (0.39, 3.10) | 0.46 |
| Related to target vessel | 13.6% (12) | 22.8% (11) | 0.55 (0.24, 1.25) | 9.4% (10) | 6.4% (3) | 1.43 (0.39, 5.20) | 0.22 |
| Not related to target vessel | 4.8% (4) | 0.0% (0) | NA | 2.9% (3) | 4.4% (2) | 0.62 (0.10, 3.68) | 0.99 |
| **All death** | 6.8% (6) | 4.4% (2) | 1.65 (0.33, 8.16) | 8.2% (9) | 6.3% (3) | 1.25 (0.34, 4.63) | 0.80 |
| Cardiac | 6.8% (6) | 2.3% (1) | 3.29 (0.40, 27.33) | 4.6% (5) | 2.1% (1) | 2.09 (0.24, 17.92) | 0.77 |
| Noncardiac | 0.0% (0) | 2.1% (1) | 0.00 (NA, NA) | 3.8% (4) | 4.2% (2) | 0.83 (0.15, 4.56) | 0.99 |
| **Definite or probable stent thrombosis** | 0.0% (0) | 6.2% (3) | - | 0.0% (0) | 2.2% (1) | - | 1.00 |

For all endpoints, percentage and (95% CI) values indicate Kaplan-Meier estimates.

Data are presented as % (n) unless otherwise noted.

Abbreviations: CABG, coronary artery bypass graft surgery; CI, confidence interval; MI, myocardial infarction; NA, not available; PCI, percutaneous coronary intervention; Undef, undefined.

**Supplemental Table 5. Two-year clinical outcomes (time-to-first event analysis) after paclitaxel-coated vs uncoated balloon angioplasty in patients with multilayer in-stent restenosis with vs without diabetes**

| 2-Year outcomes | Diabetes | | | | No Diabetes | | | | *P* for interaction |
| --- | --- | --- | --- | --- | --- | --- | --- | --- | --- |
|  | Paclitaxel-coated balloon  (N=91) | Uncoated balloon  (N=43) | HR  (95% CI) | *P* value | Paclitaxel-coated balloon  (N=85) | Uncoated balloon  (N=39) | HR  (95% CI) | *P* value |  |
| TLF | 35.2% (31) | 53.7% (23) | 0.54  (0.32, 0.93) | 0.03 | 35.3% (29) | 33.4% (13) | 0.99  (0.51, 1.90) | 0.96 | 0.16 |
| TLR | 25.0% (21) | 47.3% (20) | 0.42  (0.23, 0.78) | 0.004 | 30.3% (24) | 33.4% (13) | 0.80  (0.41, 1.57) | 0.51 | 0.15 |
| TV-MI | 13.4% (11) | 28.1% (12) | 0.41  (0.18, 0.93) | 0.03 | 6.2% (5) | 7.7% (3) | 0.78  (0.19, 3.26) | 0.73 | 0.44 |
| Cardiac Death | 9.1% (8) | 4.8% (2) | 2.01  (0.43, 9.46) | 0.37 | 3.6% (3) | 0.0% (0) | NA | 0.23 | 0.99 |

P Value is calculated using Log-Rank test.

Hazard Ratio is calculated using Cox proportional hazard regression model.
